# Biochemical evidence of epicuticular wax compounds involved in cotton-whitefly interaction

**DOI:** 10.1371/journal.pone.0250902

**Published:** 2021-05-04

**Authors:** Muhammad Azam Ali, Muhammad Azmat Ullah Khan, Abdul Qayyum Rao, Adnan Iqbal, Salah ud Din, Ahmad Ali Shahid

**Affiliations:** 1 Center of Excellence in Molecular Biology (CEMB), University of the Punjab, Lahore, Pakistan; 2 Department of Biochemistry and Biotechnology, University of Gujrat, Gujrat, Pakistan; Nigde Omer Halisdemir University, TURKEY

## Abstract

Sucking insects require a surface of plants on which the legs and the eggs of insects will adhere and to which insect mouthparts will access. The primary plant protection against insects is their surface property, which hinders the attachment of the insect’s legs and eggs. The epicuticular waxes chemistry influences the fine structure of the cuticular surface. In current study, an attempt was made to investigate the variation of chemical compounds in epicuticular waxes of four cotton species that classify them resistant or susceptible i.e., *Gossypium abroreum*, *G*. *hirsutum*, *G*. *arboreum* wax deficient mutant (GaWM3) and *G*. *harknessi* which were evaluated for their interaction with whitefly and CLCuV transmission. *Gossypium hirsutum* an insect and CLCuV susceptible cotton variety, was found to have four compounds namely Trichloroacetic acid, hexadecylester, P-xylenolpthalein, 2-cyclopentene-1-ol, 1-phenyl-and Phenol, 2,5-bis [1,1- dimethyl] which could interact with chitin of whitefly while only two compounds in *Gossypium arboreum* an insect and CLCuV resistant cotton variety could interact with chitin of whitefly. Similarly, GaWM3 and *Gossypium harkasnessi* were found to have only a single compound. Number of whiteflies found on leaves of *G*. *hirsutum* was much higher as compared to other cotton species. Keeping this fact in mind a wax biosynthetic gene CER3, from *Arabidopsis thaliana* was transformed into *G*. *hirsutum* and the plants were evaluated for their resistance against whitefly and CLCuV transmission. In microscopic analysis transgenic plants clearly showed higher amounts of leaf waxes as compared to non-transgenics. The least whitefly population and CLCuV titer of <10,000 units was found in transgenic plants compared to non-transgenic cotton where it was ≈4.5X106 units that confirmed the role of wax in insect interaction and ultimately to CLCuV transmission. This study provides novel insight on wax related compounds involved in cotton-whitefly interaction, which potentially can help in developing more efficient control strategies for this destructive pest.

## Introduction

The first zone to which approaching insects come in contact with plant is the plant cuticular surface. The physical and chemical characteristics of the epicuticular waxes can hinder the insect’s locomotion and prevent insects’ access to different plant parts. The plant cuticular compounds are responsible for initiating rejection behavior as e.g., Aphid settling to plant has been reported to be prevented by 1-hexacosanol and short–chain fatty acids and by carboxylic acids and wax esters that have antixenotic properties (C3–C13) thereby discouraging insects feeding [1]. Reduction in insect biting along with increased walking time of caterpillars and Lepidopteran insects has been found on cruciferous host leaves having long chain alcohols and amyrins in glossy-breeding wax lines [2]. The plant surface waxy compounds, including long-chain alkanes, alcohols, carboxylic acids, as well as secondary metabolites such as quinones and flavonoids, have their role in insect feeding stimulation [3]. Various approaches to investigate the role of plant epicuticular waxes on insect’s behavior has been employed. The testing of wax mutants along with genotype of plant species having different wax composition and chemistry are used to determine the influence of plant surface characteristics on insects and fungus attachment has been largely employed and still need to be done on a large scale [4]. The leaf waxes have been investigated by using the decoction method of wax extraction in which plants leaves are briefly immerged in organic solvent then this organic solvent is evaporated to concentrate the waxes [5]. For this experiment, Chloroform–methanol extraction method for wax extraction was used as reported by [6]. Modern biochemistry techniques like Gas chromatography-Mass spectrometry are used to investigate the composition of extracted waxes [7].

Epicuticular waxes in cotton are of different types and variation has been reported from species to species in terms of amount and composition [8]. Among these Asiatic cotton or *Gossypium arboreum* is well documented as resistant against CLCuD, not a single variety of *Gossypium hirsutum* has been reported as resistant against CLCuD [9]. Barozai and Husnain (2014) have reported a higher amount of wax in *G*. *arboreum* than *G*. *hirsutum* [10, 11]. Similarly, a negative correlation between CLCuV and amount of epicuticle wax has been reported in a study on different cotton varieties while a positive correlation between whitefly population and CLCuV [10]. The cotton species may be diploid or tetrapoloid consisting of A or D genome, making their physical properties different from each other [12]. Though the major role of epicuticular waxes is the provision of defense against abiotic stresses, they also play a major role in plant-insect attachment through the tarsi of insects [13]. Eigenbrode and Espelie, 1995 showed increased insect attachment with a decreased quantity of epicuticular waxes in plants [13]. Sometimes wax crystals increase the support for the insect’s attachment by providing adhesive forces to the insect tarsi [13]. In certain cases, wax crystals are decanted when insect tries to attach and hence surface becomes slippery for insects [14]. Some studies have shown that the specific compounds are involved in driving the insect behavior towards plant interaction like Glucosinolates, the secondary metabolites which affect the insect-plant interactions in Brassicaceae family [15]. So, there is a cross talk between insects and different wax types, quantity and components on the epicuticle leaf surface.

CER3 is a key wax biosynthetic gene that belongs to the *Eceriferum* family and is highly expressed during Arabidopsis’s cuticle development [16]. CER3 plays a major role in the essential alkane-forming pathway in the synthesis of major wax components [17]. CER3 and some other wax related mutant strains of *Arabidopsis thaliana* have been reported to show severe defects in epicuticle waxes and cutin biosynthesis and displayed a significantly lower level of various wax constituents [18]. CER3 and CER1 are identified as a core component of very-long-chain alkane synthesis complex when alkane biosynthesis was reconstructed in Yeast and *Arabidopsis* [19]. The long-chain fatty acids and primary alcohols are found to be major constituents of epicuticular waxes [20]. Several wax related genes have been cloned and characterized but four of these CER2, CER3, GL2, and GL15 encode regulatory loci [21].

The main objective of the current study was to determine the qualitative role of the wax compounds in the attachment of the whitefly and its ability to transmit CLCuV. Among different species of cotton *G*. *hirsutum* is most susceptible to CLCuV [22]. *G*. *hirsutum* is the most widely grown species of cotton accounting more than 90% of cotton cultivated worldwide and is the most important fibre crop globally [23]. Considering the fact, a wax biosynthesis gene was planned to be expressed in *G*. *hirsutum* for its characterization of wax quantity to determine its role in insect attraction along with using four different cotton species with varied wax contents in determining the insect visit and virus transmission.

## Material and methods

Cotton species *Gossypium abroreum*, *G*. *hirsutum*, *G*. *arboreum* wax deficient mutant (GaWM3) and *G*. *harknessi* were subjected to isolation of wax compounds by following the method mentioned below.

### Isolation of epicuticular wax

The surface area of the cotton leaf was calculated by using ImageJ [24] Easy Leaf Area Method. For wax isolation, 500mg of cotton leaves from six weeks old plants of four cotton genotypes were taken and immersed immediately in chloroform for 20 seconds at room temperature. Total 20ug of tetracosane (C24) was used as the internal standard in 20ml extract of cuticular wax. The solvent was evaporated until 1ml was left and was transferred to a new 2ml tube. Total 10 drops of diazomethane were added when the sample was dried for methylation of the free acids. Total 100ul of each of pyridine and acetic anhydride was added and the samples were kept at 60°C for 1 hour. The solvent was evaporated again, and the extract was dissolved in 500ul of heptane: toluene (1:1 v/v) followed by washing off the solution with 400ul of 1% NaHCO3.

### Comparison of the wax biochemical composition of experimental plants

For Gas chromatograph-mass spectrometry analysis, tetracosane (10 μg/mL) was added in the sample as an internal control. Quantification of single compounds was done against internal standard through manual integration of the peak areas [25].

The epicuticular wax biochemical analysis of three cotton genotypes along with wax mutant of *G*. *arboreum* showed much variation in compounds composition. Although these plants belong to the same genus, the species demonstrates a different resistance level against whitefly infestation. Some compounds are unique to a particular genotype, but many compounds are common in all genotypes. The comparison of these chemical compounds is shown in S1 Table (supplementary data). Chitin of whitefly is the first point of contact with epicuticular waxes of cotton plants [26] and structure of chitin was taken from [27].

### *Agrobacterium*-mediated genetic transformation of *Gossypium hirsutum*

Seeds of an approved local cotton (*Gossypium hirsutum*) variety CEMB-66 were taken from research repository of Centre of Excellence in Molecular Biology, University of the Punjab, Lahore, Pakistan for transformation experiments owing to its susceptibility against CLCuV and higher germination rate. Codon optimized CER3 gene was ligated in pCAMBIA1302 under CaMV35S promoter. Cotton seeds were transformed by Agrobacterium-mediated shoot apex transformation method as reported earlier [28]. Putative transgenic cotton plants which survived on media containing different hormones [29] and selection drug kanamycin (50 μg/ml) were acclimatized in pots and subjected to molecular analysis for confirmation of successful introduction of a transgene. These putative transgenic cotton plants were shifted to field containment for further analysis.

### Confirmation of transgene in putative transgenic cotton plants

Freshly emerged leaves of putative transgenic and control cotton plants were taken and subjected to DNA extraction by CTAB extraction protocol [30]. Polymerase chain reaction (PCR) was done to confirm transgene introduction by using gene-specific forward primer 5’ CACTTAGGATCTCTAGTCCAC 3’ and reverse primer 5’ GTTAGACTCATCCTTACCTCTC 3’ to amplify a fragment of 452bp length. PCR condition was set as initial denaturation at 95°C for 5 minutes followed by 35 cycles of 95°C for 30 seconds, annealing at 60°C for 40 seconds, extension at 72°C for 45 seconds. Final extension was set at 72°C for 10 minutes.

### Measurement of viral titer (β-satellite) by quantitative real-time PCR

The absolute quantification of CLCuV β-satellite components in transgenic and control cotton plants was done by Real-time PCR, the standards were prepared by serial dilution after obtaining purified plasmid having a concentration of 2.5x10^8^ molecules/μl, from Plant transformation lab of CEMB, PU, Lahore, Pakistan. A total of four serial dilutions were prepared for the standard curve in a ratio of 1:10, 1:100, 1:1000 and 1:10,000. Primers F; 5’ TTCCTATTCGCATACAACGG 3’, and R; 5’ ATGCATT GCTGGTTTGTGTT 3’ were used for absolute quantification of β-satellite titers in leaves samples by following the procedure reported earlier [31]. DNA extracted from transgenic and control cotton lines was used as template and the data sample was treated in 3 replicates. GAPDH was used as an internal control and five standards A1, A2, A3, A4 and A5 of valued 2.5x10^8^, 2.5x10^7^, 2.5x10^6^, 2.5x10^5^ and 2.5x10^4^ respectively, were used to generate standard curve. The reaction conditions for β-satellites were as follow: initial denaturation at 95°C for 5 min followed by 40 cycles of denaturation at 95°C for 30 sec, annealing at 58°C for 30 sec, and extension at 72°C for 30 sec and final elongation step at 72°C for 10 min.

### Determination of plant insect interaction (cotton plant vs whitefly)

Whiteflies were allowed to feed on experimental cotton plants for the whole season. Data was collected randomly for consecutive seven weeks and the average number of whiteflies that visited *G*. *hirsutum*, *G*. *harknessii*, *GaWM3* and *G*. *arboretum* were calculated for the whole season. The attraction of whitefly on transgenic and control plants of *Gossypium hirsutum* was also observed.

Similarly, a leaf was detached from the susceptible *Gossypium hirsutum* and its transgenic version and whiteflies were allowed to feed on them. Number of adult whiteflies along with eagling and whitefly nymph were compared in transgenic and control.

### Comparison of transgenic and control leaf cross sections by microscopy

Leaves from transgenic control cotton (*G*. *hirsutum*) and *G*. *arboreum* (high wax CLCuV resistant Asiatic cotton) plants were excised and sections of 2 to 3 mm were cut with a razor blade and fixed in Paraformaldehyde Fixative. Samples were embedded in molten paraffin and were allowed to settle at room temperature then proceeded for microtomy. Tissue sections of 5–10μm were cut with Microtome (Microme HM 340-E). Before proceeding to microscopy embedded sections were dried at 37°C for one hour and both transgenic and control samples were subjected to dewaxing for better conclusive imaging. Samples were kept at 56°C for 10 minutes and then slides were dipped into xylene three times for 5 minutes each with new xylene every time for complete removal of wax from the cuticle. Images were taken through inverted fluorescent (Olympus1X51) microscope (200x magnification).

## Results

### Comparative alignment of identified wax compounds of cotton species

These compounds were aligned using PyMOL software version 2.3 (https://pymol.org/2/) which revealed a different structure for each genotype (supplementary file).

### Determination of cotton wax compounds interaction with whitefly chitin

#### Trichloroacetic acid, hexadecylester

**Fig 1:**
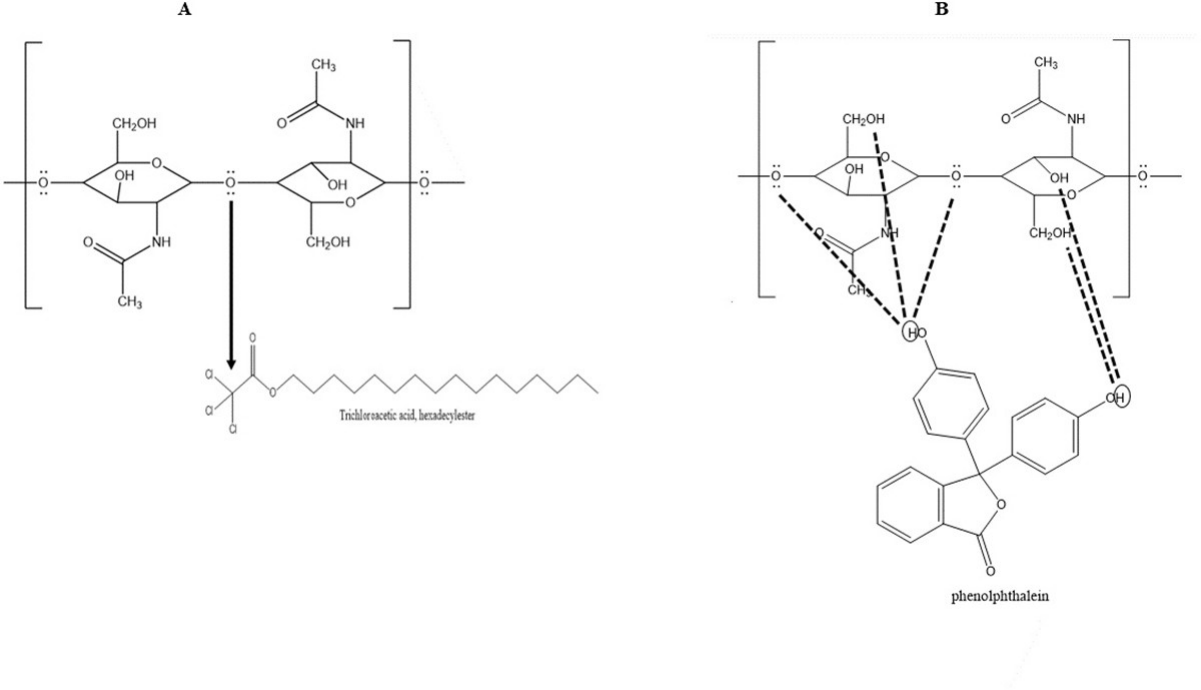
**(A)** Interaction of Trichloroacetic acid, hexadecylester with chitin where the central carbon attached to chlorine is highly deficient to the electron and can interact with the electron pair from chitin. **(B)** Interaction of P-xylenolpthalein with chitin by making hydrogen bonds.

Trichloroacetic acid, hexadecylester were identified in the wax composition of *G*. *hirsutum*. The chlorine atoms were found to be attached with the central carbon attached to the carbonyl group thus the central carbon is highly deficient to the electron chain and shows some affinity to the chitin [32] (Fig 1A).

#### P-xylenolpthalein

The P-xylenolpthalein was also one of the identified compounds in wax composition of *G*. *hirsutum* having the similarity in structure with phenolphthalein. The OH group of phenolphthalein have the affinity to attract electron. They can make a hydrogen bond with oxygen atom present between the polymers or any other OH group of chitin upon insect visit (Fig 1B).

#### 2-cyclopentene-1-ol, 1-phenyl-

**Fig 2:**
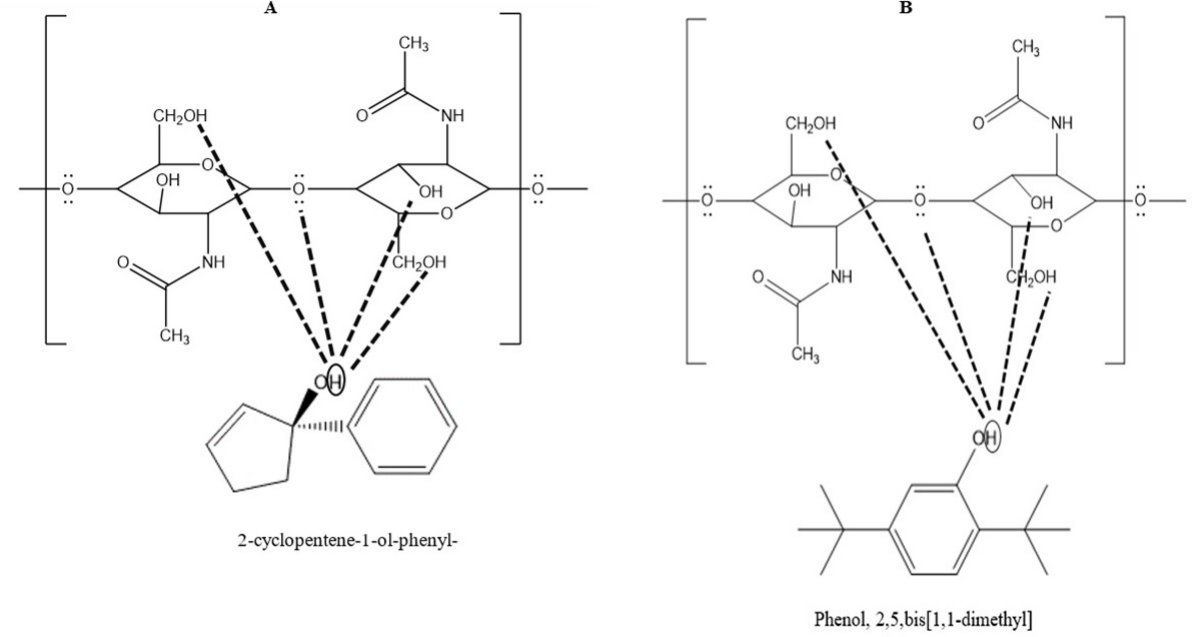
**(A)** Interaction of hydrogen of 2-cyclopentene-1-ol-phenyl- with oxygen and hydroxyl group present in chitin. **(B)** Interaction of hydrogen of Phenol,2, 5, bis [1,1-dimethyl] with oxygen and hydroxyl group present in chitin.

This compound is present both in *G*. *hirsutum* and *G*. *arboreum*. The H in the 2-cyclopentene-1-ol, 1-phenyl- is above the board structure that interacts with Oxygen present between the polymers and OH groups present in the chitin structure [33] (Fig 2A).

#### Phenol, 2,5-bis [1,1- dimethyl]

This compound is present in *G*. *arboreum*, GaWM3 and *G*. *hirsutum*. The Phenol, 2,5-bis [1,1- dimethyl] is electron deficient in OH group that can interact with ‘O’ present between the polymers and OH groups present in the chitin structure (Fig 2B).

#### Piperidinone, n-|4-bromo-n-butyl|

**Fig 3:**
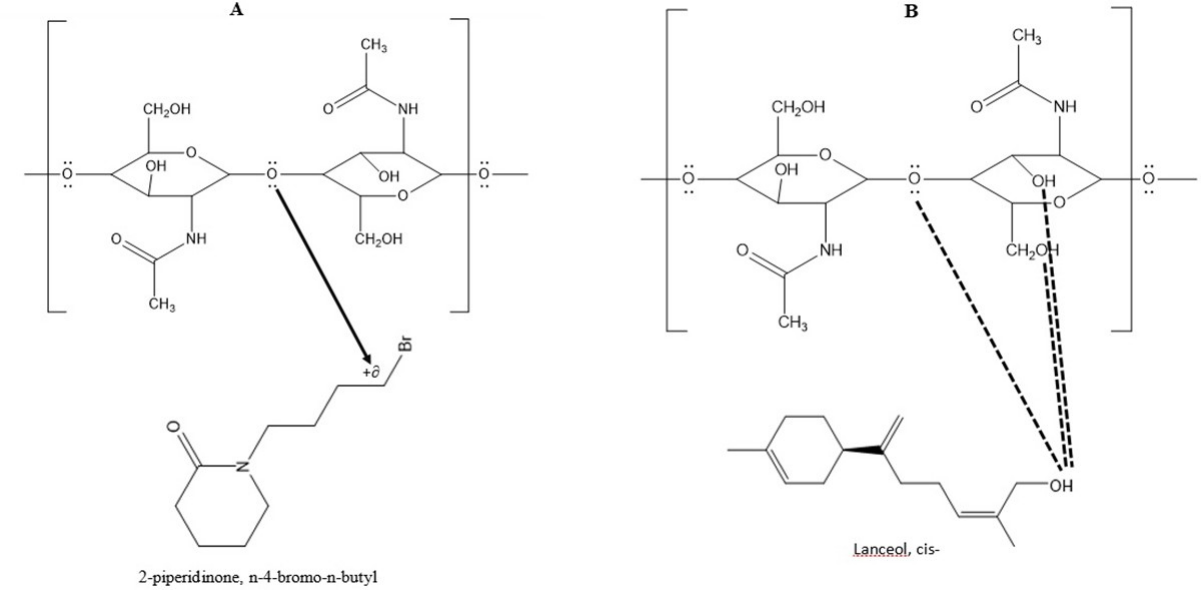
**(A)** Interaction of piperidinone, n-|4-bromo-n-butyl| with chitin of whitefly **(B)** Interaction of hydrogen of Lanceol, cis- with oxygen and hydroxyl group present in chitin.

This unique compound was found only in wax of *G*. *arboretum*. The carbon attached to Br is partially positive and has the affinity for the electron pair present in the “O” of the chitin (Fig 3A).

#### Lanceol, cis-

This compound was found in wax of *G*. *harknessii*. The H present in OH group is electron deficient which is above the board and can interact with O present in chitin [34] (Fig 3B).

### Compounds of *G*. *hirsutum* that are unlikely to interact with chitin

The compounds Tetradecane, 2,6,10-trimethyl-, 1,2-Benzenedicarboxylic acid, di-isooctyl ester and Nonadecane are unlikely to interact with chitin however 2-Trifluoroacetoxyteradecane interact with chitin but the group have the carbon with F and unlikely to interact with chitin.

### Compounds of *G*. *arboreum* that are unlikely to interact with chitin

The wax compounds present in *G*. *arboreum* are unlikely or very difficult to interact with chitin because of the structure or chemical composition i.e., 1,2-Benzenedicarboxylic acid, diisooctyl ester, 3-trifluoroacetoxytetradecane, 6-Octadecenoic acid, methyl ester, Heptadecanoic acid, 16-methyl-, methyl ester, 4-heptafluorobutyroxypentadecane, Methoxyacetic acid, 2- tridecylester, Nonadecane, Silane, trichlorodocosyl- and Tetradecane, 2,6,10-trimethyl.

In Silane, trichlorodocosyl- Si has 3 Cl but is less negative than carbon. In 4-heptafluorobutyroxypentadecane, the carbon having 7 F is below the board and has electron cloud of F. Similarly, 3-trifluoroacetoxytetradecane the carbon is below the plane so it is very unlikely that these compounds along with rest of the compounds mentioned above will interact with chitin.

### Compounds of GaWM3 that are unlikely to interact with Chitin

The compounds of GaWM3 P-Xylenolpthalein, Trichloroacetic acid, hexadecylester, 2-Trifluoroacetoxyteradecane, |5-[3-methoxymethoxy-10,13-dimethyl-2,3,4,9,10,11,13,14,15,16,17-dodecahydro-, 15,17,19,21- Hexatriacontatetrayne, Octadecane, 1-|2-[hexadecyloxy]ethoxy|-, A-D-Glucopyranoside, methyl-2-[acetylamino]-2-deoxy-3-O-[trimethylsillyl]-,cyclic methyl bronate, Ethanol, 2-[octadecyloxy], 7,9-Di-tet-butyl-1-oxaspiro[4,5] deca– 6, 9-diene-2,8-dione, Diethyl phthalate, Eicosane, 2-methyl-, Eicosane, 2-methyl-, Hexadecane, α-Caryophyllene, Caryophyllene, 1,2-Benzenedicarboxylic acid, diisooctyl ester, Tetradecane, 2,6,10-trimethyl-, Methoxyacetic and Nonadecane are unlikely to interact with chitin.

### Compounds of *G*. *harknessii* that are unlikely to interact with Chitin

The compounds found in wax of *G*. *harknessii* 2-napthalenemethanol,decahydro- α, α, 4a-trimethyl-8-methylene-,|2R-[2^α^,4aα,8aβ]|-, 2,6,10-dodecatriene-1-ol,3,7,11-tromethyl-acetate,[E,E]- 2,6,10-dodecatriene-1-ol,3,7,11-tromethyl-acetate,[E,E]-, Napthalene,1,2,3,4,4a,5,6,8a-octahydro-7-methyl-4-4methylene-1-[1-methylethyl],[1α,4aβ,8aα]-, α-Caryophyllene are Caryophyllene are unlikely to interact with chitin.

### Transformation of codon optimized CER3 gene in cotton

**Fig 4:**
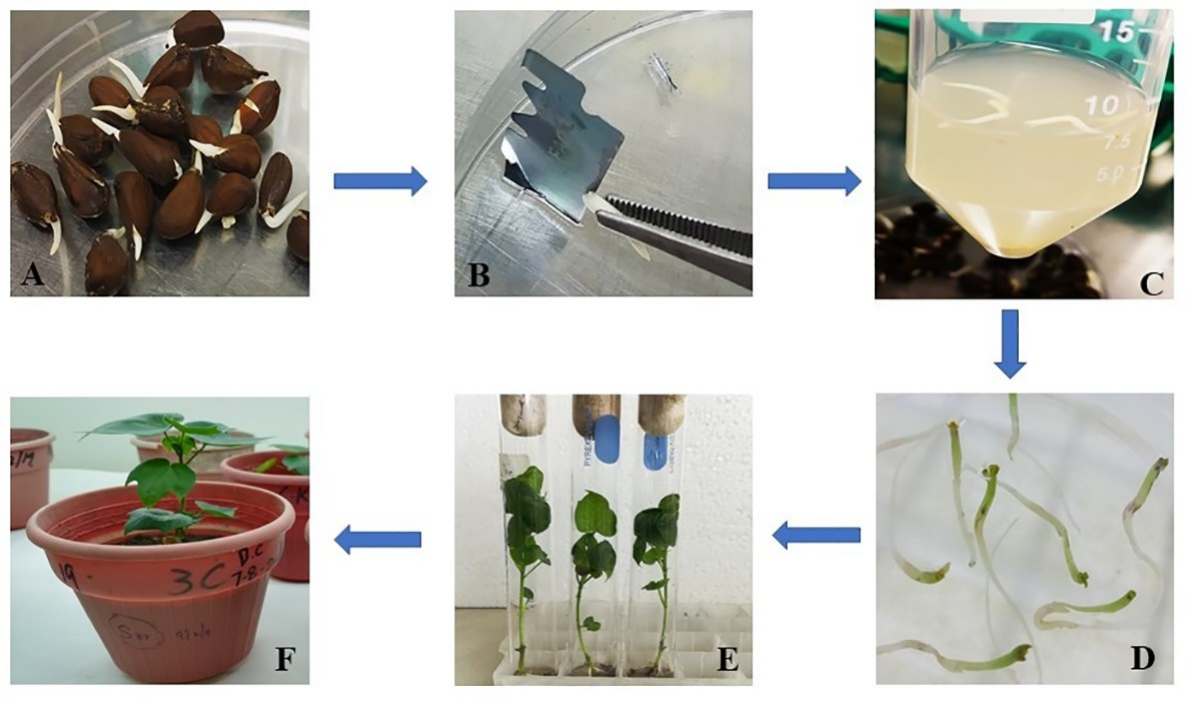
A: Germinated seeds, B: Injuring the embryos with sharp blade on shoot apex side, C: Co-cultivation of embryos with agrobacterium, D: Embryos in growth medium, E: plants in rooting and shooting media, F: Plants in soil pots for acclimatization.

A total of 6000 embryos were used in agrobacterium mediated shoot-apex transformation of cotton, and transgenes’ selection was done on selection drug kanamycin (Fig 4A–4F). After acclimatization, four of the best performing putative transgenic cotton plants were selected for further analysis. The transformation efficiency was calculated to be 0.97% based on plant survival and 0.1% based on confirmation of transgene by molecular analysis.

### Confirmation of putative transgenic plants

**Fig 5:**
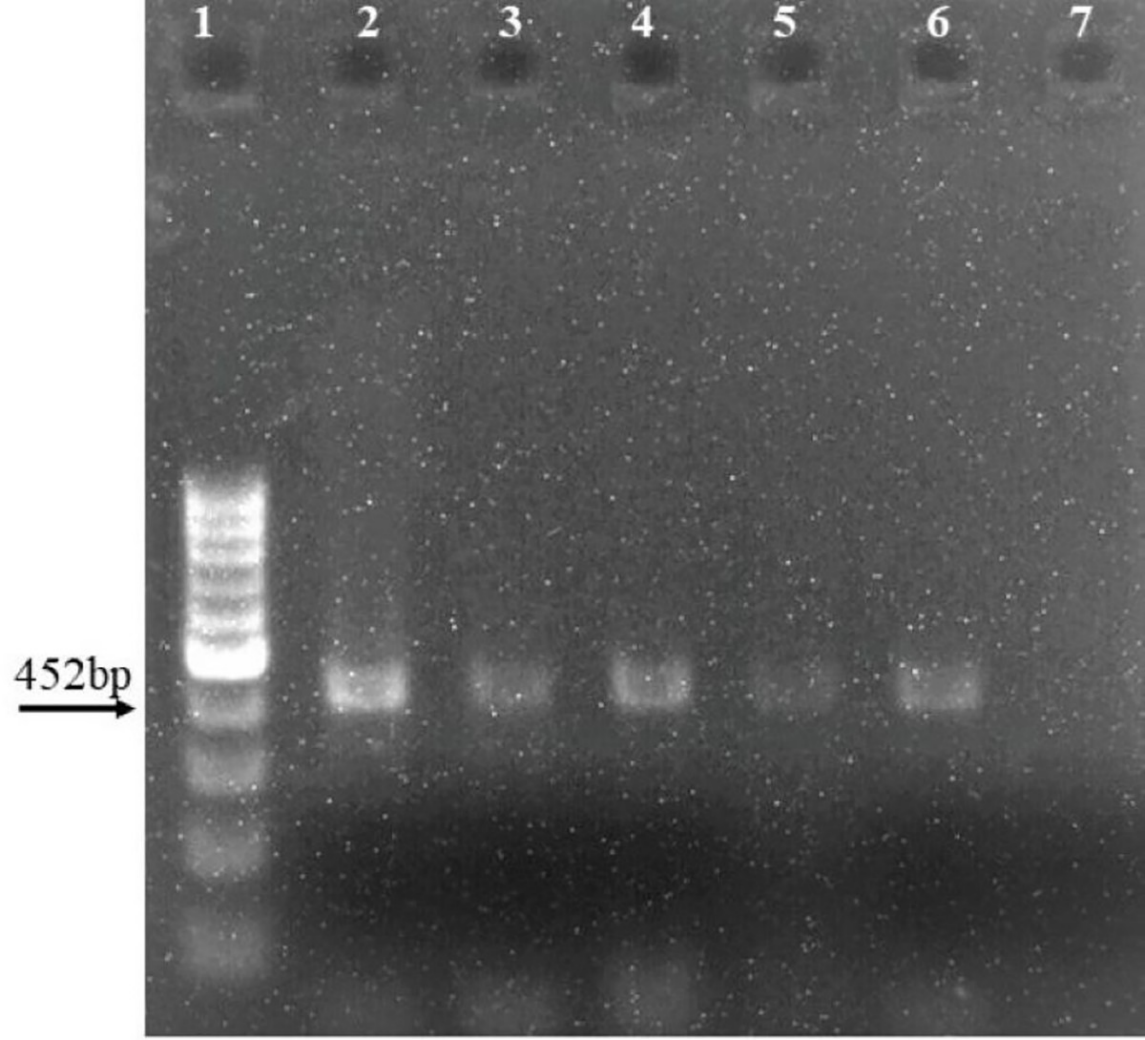
Transgene (CER3) amplification through PCR. Lane 1: 100bp Ladder, Lane 2: positive control, Lane 3,4,5,6 transgenic plants, Lane 7: negative control (non-transgenic cotton).

Transgene introduction into cotton plants was confirmed through amplification by using gene specific primers. A fragment of 452bp was amplified in four transgenic cotton plants, abbreviated as wax lines (WL-1, WL-2, WL-3, WL-4) and positive control, while no amplification was observed in the negative (non-transgenic) control cotton plants (Fig 5).

### Quantitative real-time PCR for β-satellite titers

**Fig 6:**
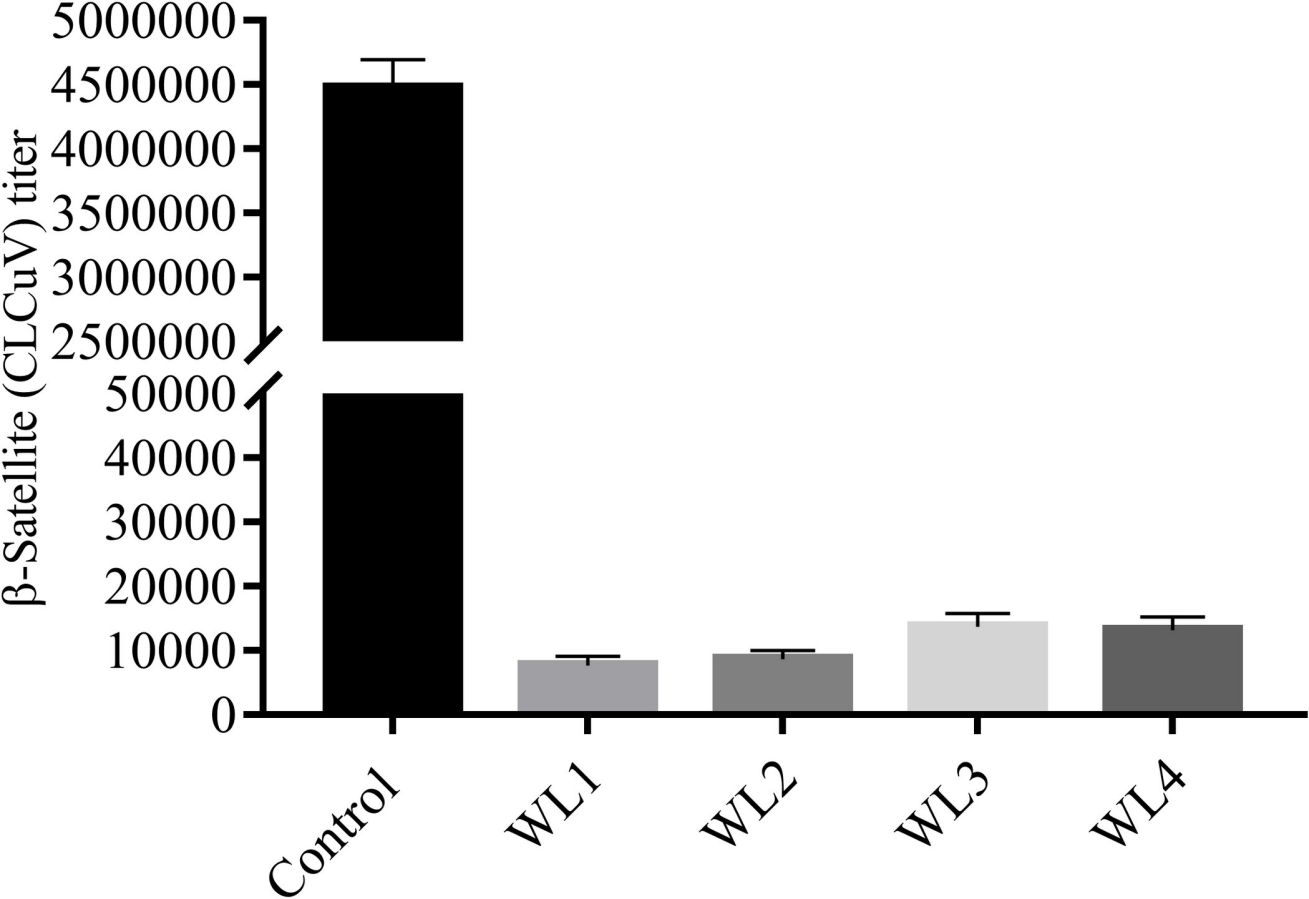
Measurement of CLCuV titer (β-satellite) in control and transgenic cotton plants (WL1, WL2, WL3, WL4).

The study’s indirect objective was to evaluate the transgenic cotton plants for CLCuV titer as the least whitefly population will result in reduction of virus copies delivered by whitefly and enable the plants to cope with fewer copies of CLCuV. The CLCuV, β-satellite components were evaluated in all four selected transgenic cotton lines, namely WL1, WL2, WL3 and WL4 in comparison to non-transgenic control cotton plants. Significantly less CLCuV titer i.e.,7738 to 13775 in cotton line WL1 and WL3 as compared to non-transgenic control cotton *i*.*e* 4.5x10^6^ was observed (Fig 6). *Gossypium hirsutum* is most susceptible to CLCuV, but transgenic plant having wax related transgene showed a significant improvement in resistance against virus transmission from whiteflies that supports the idea of wax role against insects and indirectly to virus.

### The relationship among whiteflies population and epicuticular waxes composition

**Fig 7:**
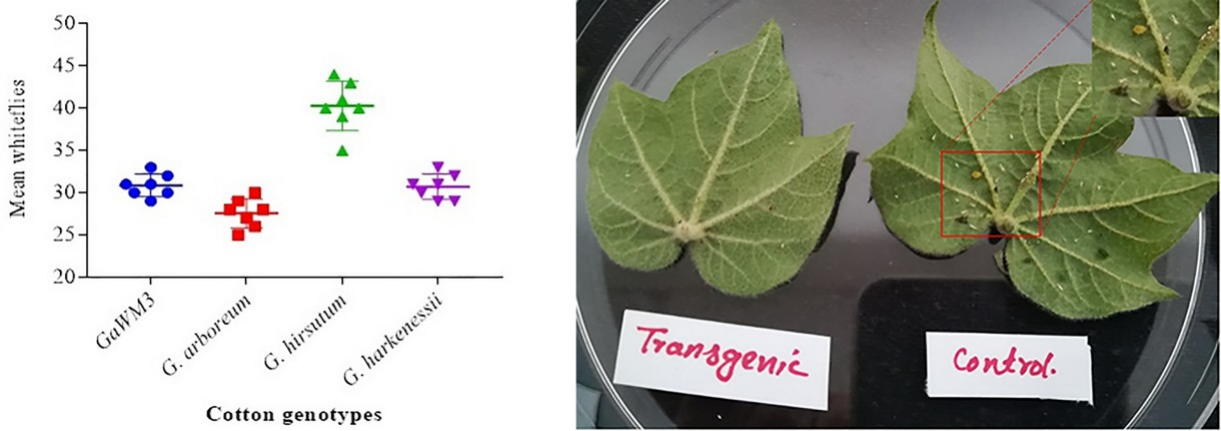
**(A)** Number of whiteflies visits to different genotypes of cotton **(B)** The trend of whitefly both on transgenic and control cotton (*G*. *hirsutum*) plants.

The average number of whiteflies calculated on *G*. *hirsutum* was 40 compared to 29 of *G*. *harknessii*, 31 of GaWM3 and 27 *G*. *arboreum*. *G*. *hirsutum* is most susceptible to CLCuV and an attractive cotton species for whiteflies. This experiment supports the data that variation in wax compounds composition has its role in determining the attraction of whiteflies for their visit to the plant. In one aspect reduction of wax quantity and change of composition in one of the mutant GaWM3 of *G*. *arboreum* a CLCuV resistant cotton variety resulted in an increased tendency of whitefly visits (Fig 7A). Feeding assay for both transgenic and control cotton plants was also done and the transgenic plants of *G*. *hirsutum* were observed to be less attractive for whitefly as compared to non-transgenic control cotton plants which further support the idea of wax role in changing the insect behavior for their visit to cotton plants (Fig 7B).

### Cuticular wax in transgenic and control leaves through microscopy

**Fig 8:**
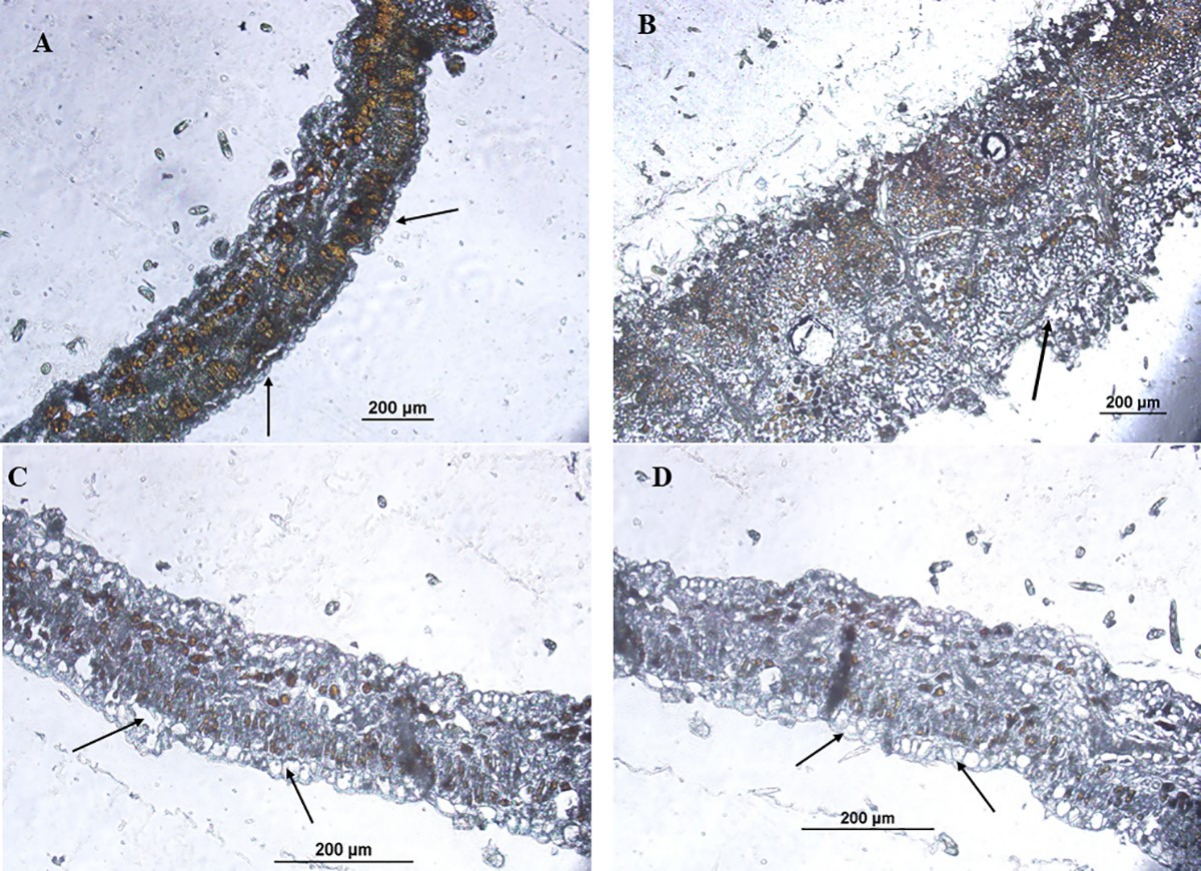
**(A):** Non-transgenic (control) *Gossypium hirustum* after wax removal. **(B):** *Gossypium arboreum* after wax removal **(C):** Transgenic *Gossypium hirustum* showing higher wax removal after treatment with the organic solvent. **(D):** Transgenic *Gossypium hirustum* showing higher wax removal after treatment with the organic solvent.

The comparison of dewaxed leaves from control (Fig 8A), transgenic *G*. *hirsutum* (Fig 8C and 8D) and *G*. *arboreum* (Fig 8B) showed a clear difference in wax contents. Significantly improved wax contents were observed in transgenic plant leaves as compared to control, while *G*. *arboreum* showed highest of the wax contents (Fig 8). The results clearly described that wax contents were improved in transgenic cotton and its relation to whitefly tendency and CLCuV titer demonstrated a direct relation between wax contents and CLCuV transmission.

## Discussion

Plant surface cuticle acts as a barrier or first line of defense against insects approaching to plant surface. The layer of plant impregnated with intracuticular waxes and epicuticular waxes is called cuticle [35]. Though the role of epicuticular waxes against insect-plant interaction has already been defined, the wax compounds’ interaction with insects that embark on the susceptivity or resistance against insects has not been explored.

Cotton leaf curl disease (CLCuD) caused by CLCuV is a serious disease of cotton and several other malvaceous plant species transmitted by the whitefly *Bemisia tabaci*. No variety of *Gossypium hirsutum* has been reported as resistant against devastating virus whose carrier is whitefly [9]. This disease was reported in Pakistan and India but gradually it has spread to other cotton growing areas of the world, especially south China. However, Asiatic cotton (*Gossypium aroboreum*) is resistant to this disease. Although the exact mechanism is not well defined, higher amounts of epicuticular waxes have been reported as one of the key barriers for whiteflies in *Gossypium aroboreum* [11, 31, 36]. Khan et al. 2015 reported that different cotton species have variable quantity of waxcontents and variation in their response in terms of virus resistance and susceptibility [31].

Keeping in mind the importance, an attempt was made to determine the difference of wax compounds in four different types of cotton genotypes and further a wax biosynthesis gene CER3 from *Arabidopsis thaliana* was transformed in *Gossypium hirsutum* to find out whether increased epicuticular wax amount can reduce the foraging success of whitefly and ultimately CLCuV titer (Fig 6). Waxes from four different species of cotton were isolated by decoction method as reported by [5]. Wax compounds were aligned by using PyMOL version 2.3 as reported by [37] to see the difference (supplementary data) and compounds were further identified by GC-MS (supplementary data) and comparison was made among wax compounds of all the four genotypes. Being more susceptible to whitefly, *G*. *hirsutum* showed the presence of five different compounds i.e., Trichloroacetic acid, hexadecylester, P-xylenolpthalein, 2-cyclopentene-1-ol, 1-phenyl-, and Phenol, 2,5-bis [1,1- dimethyl], which showed a bonding with chitin of whitefly (Figs 1 and 2). Fig 7A shows the number of visits of whitefly on different species of cotton under the same conditions, which was calculated to be highest for *G*. *hirsutum*. Though the exact mechanism of insect interaction and repulsion is not cleared yet in more depth, the evidence obtained has proved that increased epicuticular waxes make it less attractive for insects [38, 39]. The transgenic plants of *Gossypium hirsutum* with improved epicuticular waxes showed a greater resistance against insects and were found to be less attractive for whitefly as compared to its non-transgenic version (Fig 7B) which supports the idea that improved wax contents make it less attractive for the whitefly. To compare wax contents in transgenic and control plant, waxes were removed from leaf cross sections by using organic solvent xylene and significantly improved wax contents were observed in transgenics as compared to control (Fig 8). Apart from being less attractive, the foraging success and virus transmission ability of whitefly were also significantly reduced. The amount of wax and the wax compounds composition data supports the idea that certain compounds that make the whitefly attraction towards *G*. *hirsutum* and improved wax contents provide a significant resistance against whitefly.

We studied the cross-talk between the whitefly and leaf epicuticle while comparing transgenic lines with improved wax contents with a non-transgenic version of Gossypium hirsutum. The results demonstrated significantly low viral titer in *Gossypium hirsutum* with increased wax contents as compared to control lines. *Gossypium hirsutum* is susceptible against whitefly and CLCuV [40], but from our results, we can demonstrate that improved wax contents can make it resistant against whitefly and CLCuV.

## Conclusion

The study demonstrated that not only the quantity but also the composition of wax contents have their role in defining the nature of plants as susceptible or resistant as well provision of attraction to insects or least interest for insects to visit the plant which indirectly influence the infections transferred by insects to plants like in case of CLCuV titer.

## Supporting information

S1 Fig Alignment of identified wax compounds of cotton species. (A) *G*. *arboreum* (B) GaWM3 (C*) G*. *hirsutum* (D) *G*.*harknessii*.
(JPG)

S1 Table List of wax compounds. (PDF)

S1 Raw image (PDF)
