# Supplementary figures and images for: Biochemical evidence of epicuticular wax compounds involved in cotton-whitefly interaction
Source: PLoS One. 2021 May 4;16(5):e0250902. doi: 10.1371/journal.pone.0250902 (PMC8096116; doi:10.1371/journal.pone.0250902)

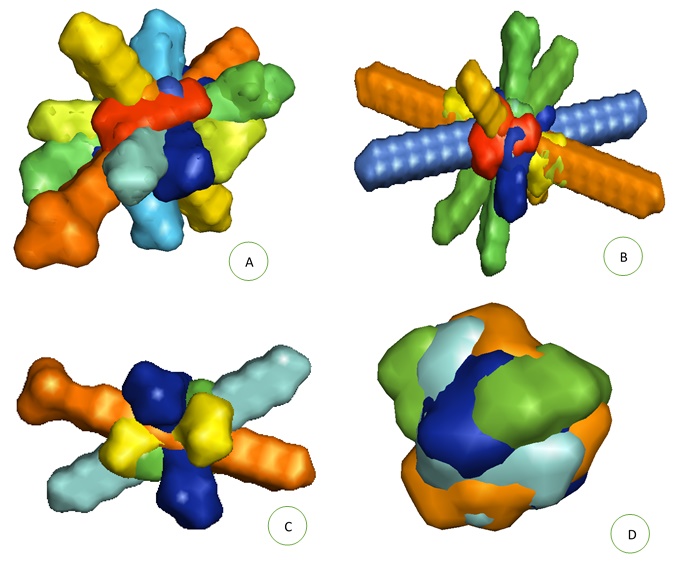

Supplement: S1 Fig — (A) G. arboreum (B) GaWM3 (C) G. hirsutum (D) G.harknessii. (JPG) [file pone.0250902.s001.jpg]

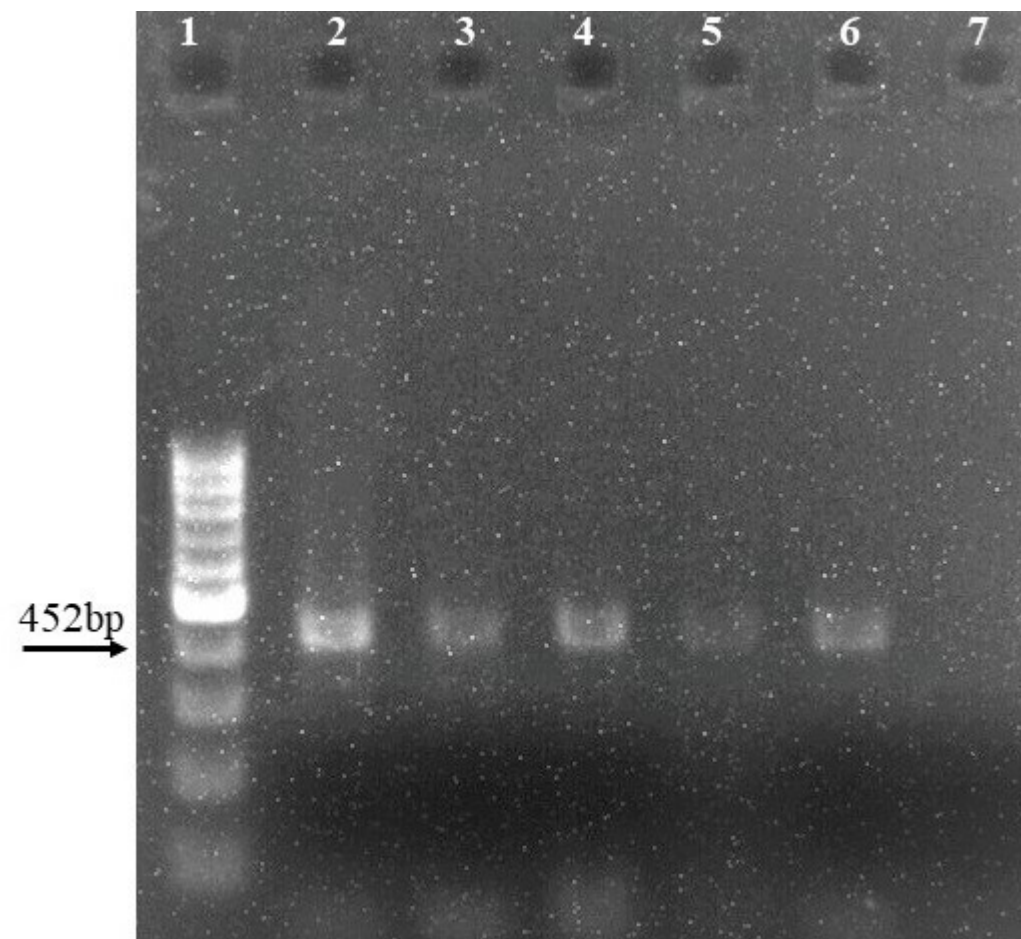

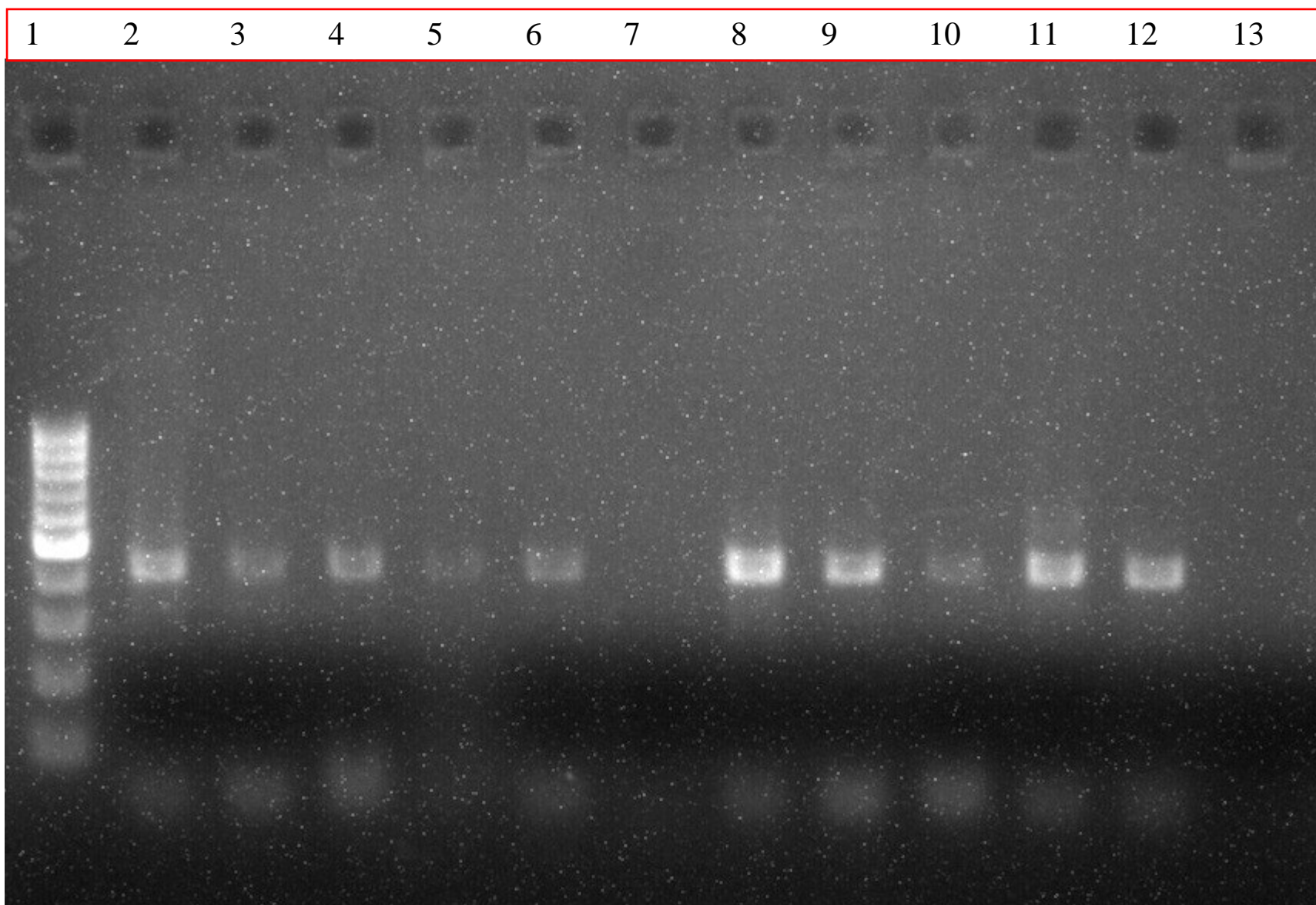

Supplement: S1 Raw image — (PDF) [file pone.0250902.s003.pdf]
