# Supplementary material for: Biochemical evidence of epicuticular wax compounds involved in cotton-whitefly interaction
Source: PLoS One. 2021 May 4;16(5):e0250902. doi: 10.1371/journal.pone.0250902 (PMC8096116; doi:10.1371/journal.pone.0250902)
Supplement: S1 Table — (PDF) [file pone.0250902.s002.pdf]

Biochemical Compounds	*Gossypium arboretum*	GaWM3	*Gossypium hirsutum*	*Gossypium harknessii*
2-cyclopentene-1-ol, 1-phenyl-	+	-	+	-
Nonadecane	+	+	+	-
3-trifluoroacetoxytetradecane	+	-	-	-
Phenol, 2,5-bis [1,1- dimethyl]	+	+	+	-
Methoxyacetic acid, 2- tridecylester	+	+	-	-
2-piperidinone, n-|4-bromo-n-butyl|	+	-	-	-
4-heptafluorobutyroxypentadecane	+	-	-	-
Tetradecane, 2,6,10-trimethyl-	+	+	+	-
Silane, trichlorodocosyl-	+	-	-	-
6-Octadecenoic acid, methyl ester	+	-	-	-
Heptadecanoic acid, 16-methyl-, methyl ester	+	-	-	-
1,2-Benzenedicarboxylic acid, diisooctyl ester	+	+	+	-
Caryophyllene	-	+	-	+
α-Caryophyllene	-	+	-	+
Hexadecane	-	+	-	-
Eicosane, 2-methyl-	-	+	-	-
Diethyl phthalate	-	+	-	-
7,9-Di-tet-butyl-1-oxaspiro[4,5] deca – 6, 9-diene-2,8-dione	-	+	-	-
Ethanol, 2-[octadecyloxy]	-	+	-	-
A-D-Glucopyranoside, methyl-2-[acetylamino]-2-deoxy-3-O-[trimethylsillyl]-,cyclic methyl bronate	-	+	-	-
Octadecane, 1-|2-[hexadecyloxy]ethoxy|-	-	+	-	-
15,17,19,21- Hexatriacontatetrayne	-	+	-	-
|5-[3-methoxymethoxy-10,13-dimethyl-2,3,4,9,10,11,13,14,15,16,17-dodecahydro-1Hcyclopenta|a|[phenanthren-17-yl]-hex-1-ynul|-trime	-	+	-	-
2-Trifluoroacetoxyteradecane	-	-	+	-
Trichloroacetic acid, hexadecylester	-	-	+	-
P-Xylenolpthalein	-	-	+	-
Lanceol, cis-	-	-	-	+
Napthalene,1,2,3,4,4a,5,6,8a-octahydro-7-methyl-4-4methylene-1-[1-methylethyl],[1α,4aβ,8aα]-	-	-	-	+
2,6,10-dodecatriene-1-ol,3,7,11-tromethyl-acetate,[E,E]- 2,6,10-dodecatriene-1-ol,3,7,11-tromethyl-acetate,[E,E]-	-	-	-	+
2-napthalenemethanol,decahydro- α, α, 4a-trimethyl-8-methylene-,|2R-[2^{α},4aα,8aβ]|-	-	-	-	+
